# Alendronate Sodium as Enteric Coated Solid Lipid Nanoparticles; Preparation, Optimization, and *In Vivo* Evaluation to Enhance Its Oral Bioavailability

**DOI:** 10.1371/journal.pone.0154926

**Published:** 2016-05-05

**Authors:** Khaled Mohamed Hosny

**Affiliations:** 1 Department of Pharmaceutics and Industrial Pharmacy, Faculty of Pharmacy, King Abdulaziz University, Jeddah, Saudi Arabia; 2 Department of Pharmaceutics and Industrial Pharmacy, Faculty of Pharmacy, Beni Suef University, Beni Suef, Egypt; University of Helsinki, FINLAND

## Abstract

Treatment of osteoporosis with alendronate sodium has several challenges. The first challenge is the low bioavailability. The second main challenge is side effects, which include oesophageal ulceration. The aim of this research was to reformulate alendronate sodium as enteric coated solid lipid nanoparticles in order to enhance its bioavailability, and preventing the free alendronate sodium from coming into direct contact with the gastrointestinal mucosa, and thereby reducing the possibility of side effects. Enteric coated solid lipid nanoparticles were prepared according to the Box-Behnken design employing Design expert^®^ software, and characterized for size, morphology, and entrapment efficiency. The optimized formula was coated with an Eudragit S100 and evaluated for drug release in acidic and basic media, stability studies and pharmacokinetic evaluations on rabbits. The results indicated that, using Derringer's desirability functional tool for optimization, the highest entrapment efficiency value of 74.3% and the smallest size value of 98 nm were predicted under optimum conditions with a desirability value of 0.917. The optimized nanoparticles released alendronate sodium only at an alkaline pH. The pharmacokinetic evaluation revealed that alendronate sodium bioavailability was enhanced by more than 7.4-fold in rabbits. In conclusion, enteric coated solid lipid nanoparticles is a promising formula for the delivery of alendronate sodium, eliminating its oesophageal side effects and enhancing its bioavailability.

## Introduction

Alendronate sodium (ALS) is a nitrogen-containing oral bisphosphonate used for the treatment of osteoporosis [1]. Approximately 50% of patients treated with ALS show a decrease in the incidence of osteoporotic fractures in the hips and spine as a result of improved bone density and reduced bone resorption [2]. The first challenge for using ALS is the low bioavailability; only 0.6% of the ALS dose is absorbed when taken two hours before food or after overnight fasting as a result of ALS hydrophilicity and the negative charges that hinder crossing the lipoid biomembrane of the digestive tract [3]. The second main challenge is the side effects, which include oesophageal ulceration and erosion associated with bleeding. As a result of these constraints, using this drug requires precautions and complicated instructions, as the patient should be upright for at least 30 minutes without any food or beverages after taking each dose. Up to 60% of the patients stop taking weekly ALS during the first year as a result of its side effects and complicated instructions [4].

Solid lipid nanoparticles have recently gained interest for drug delivery applications [5]. SLNs retain the advantages of traditional liposome-based formulations, such as high absorption and biocompatibility, while overcoming issues of stability commonly encountered with liposomes [6]. Due to the simplicity of formulation and basic biocompatibility of the constituent materials, SLNs have become attractive biomaterials for the field of nanomedicine research [7]. They have the potential for targeted delivery and prolonged drug release [8], enhanced drug absorption via improvement in permeation within the GIT, and improved drug stability [9]. They also possess very low inherent toxicity due to the use of natural and physiological lipids and are amenable to large-scale production methods and economically viable [10]. SLNs also possess some limitations, such as drug expulsion [11], and a tendency to agglomerate [12]. These limitations are linked to the physicochemical properties of the SLNs (e.g., lipids, surfactants/co-surfactants) and methods used for formulation. They are advisable for the incorporation of lipophilic (poorly water soluble) and hydrophilic drugs within the lipid matrix in considerable amounts [13]. SLN tolerability and toxicity have also been characterized in vivo and in vitro [14], and they are thought to be generally well tolerated owing to their composition from physiologically similar lipids [15].

The technology of enteric coating is used for several applications to protect the drug from gastric fluids and enzymes or to protect the oesophagus and stomach from the irritant action of some drugs. An enteric coating prevents the drug in SLNs from disintegrating in the stomach and will increase the intact amount of SLNs delivered to the duodenum of the small intestine [16], which improves the bioavailability of ALS and reduces the mucosal irritant effect. The main objective of this work is to formulate ALS in the form of SLNs to improve the bioavailability of the drug. SLNs are then coated with an enteric coating material (Eudragit S100) to reduce side effects on the oesophagus and allow elderly patients to tolerate the drug easily.

## Materials and Methods

### Materials

Alendronate Sodium was kindly supplied by (Saja Pharmaceuticals Co. Ltd., Jeddah, Saudi Arabia). Glyceryl monostearate, (M.P. 52–54°C; molecular weight 358.63), Labrasol, and Compritol 888ATO were obtained as a gift from Gattefosse (France). Cremophor 25 and stearic acid were purchased from Sigma Chemical Company (St. Louis, Missouri, USA). Lutrol 68 and Lutrol 127 were obtained from Fluka Chemical Company (Buchs, Germany). Eudragit was kindly supplied by (Evonik Industries AG, Essen, Germany). All other chemicals utilized were of analytical reagent grade.

## Methods

### Solubility Study of Alendronate sodium in various lipids

ALS (10 mg) was partitioned in a mixture of melted lipid (1 g individually) (e.g., glyceryl monostearate, stearic acid, compritol 888 ATO, and Precirol ATO5) and 10 ml of hot distilled water at 70°C and shaken for 30 min in a hot water bath. The aqueous phase was separated after cooling by ultracentrifugation, and the drug concentration was assayed at 333 nm using the spectrophotometric method proposed by Al Deep et al. (2004) [17]. Among all of the lipids used, glyceryl monostearate showed the highest drug portioning; hence, it was chosen for formulation of SLNs.

### Selection of formulation and process parameters

A set of experiments was carried out in a stepwise manner to deduce the optimal formulation and process parameters for the development of ALS-loaded solid lipid nanoparticles. The selection of lipid was based on the solubility and partitioning of ALS in the different lipids. The selection of independent variables was based on a previously performed screening study, which indicated that X1 represent the % of lipid (glyceryl monostearate), X2 represent the % of surfactant (Lutrol 68), and X3 that represent the sonication time are the three factors that have more a significant effect on ALS-SLN than other factors usually studied, such as the type of surfactant (e.g., Lutrol 68, Cremophor 25, and Labrasol) and homogenization time. The dependent variables were particle size (PS) and entrapment efficiency (EE%).

The Box-Behnken experimental design is one of the response surface models used to hit the target, reduce variability in the experiment, maximize/minimize a response that increases the production yield or decreases the amount of waste, and represent opportunities for extensive financial gain. This model has an orthogonal design. The factor levels are evenly spaced and coded for low, medium and high settings: −1, 0 and +1. Therefore, the process is optimized to obtain the levels of the independent variables that provide the optimum response values. The actual value and coded value of different variables are given in Table 1. The selection of these limits was based on acceptable domains for each variable in view of a therapeutic application, and the optimization procedure was carried out within these domains.

**Table 1:** Variables and their actual and coded values selected to perform the optimization.

Level	Low	Medium	High
**Coded values**	-1	0	+1
**X1**^a^	20%	30%	40%
**X2**^a^	5%	10%	15%
**X3**^a^	2 minutes	5 minutes	8 minutes

^a^ X1 is % of lipid, X2 is % of surfactant, and X3 is sonication time

### Preparation of Alendronate sodium loaded solid lipid nanoparticles

The solvent injection technique was carried out at a temperature above the melting point of the lipid. Specific amounts of glyceryl monostearate (according to the experimental design in Table 1) were dissolved in 10 ml of ethanol and rapidly injected through an injection mode into a stirred (1000 rpm) 100 ml aqueous solution of 0.1% ALS containing a specified amount of surfactant as given in Table 1. Thereafter, the dispersion was ultracentrifuged at 20,000 rpm for 30 min at 10°C, and aggregates were purified by dialysis bag and re-suspended in 10 ml of aqueous phase containing 2.5% Lutrol 127 as a nanoparticle stabilizer with stirring at 1000 rpm for 30 min. Then, the precipitates of certain formulae were dispersed in buffer solution (pH 7.4) in which eudragit S100 was previously dissolved and sonicated for a specific time according to the experimental design (Table 1). The suspensions were rendered acidic by the addition of an acidic solution at pH 2 in order to precipitate the eudragit. The final suspensions were centrifuged for 30 minutes at 15000 rpm, and the eudragit coated ALS-SLNs were then subjected to freeze-drying [18].

### Determination of Particle size and zeta potential

Particle size and zeta potential measurements were performed using dynamic light scattering experiments at a fixed scattering angle of 173° using a Malvern Zetasizer instrument (#Nano ZS 4800, UK) at 25°C. Each measurement was performed three times, and an average value was calculated.

### Entrapment efficiency Measurement (EE%)

The percentage drug entrapment efficiency of ALS in SLN formulations was determined by centrifugation at 15,000 rpm at 10°C for 45 min. The free ALS amount present in the supernatant was determined at 333 nm by the spectrophotometric method proposed by Al Deep et al. 2004^17^.

The drug entrapment efficiencies were calculated from the following equation [19].

$$\text{\% EE = }\left[{\text{ALS}}_{\text{T}}\text{ - }\left({\text{ALS}}_{\text{S}}{\text{+ ALS}}_{\text{P}}\right)\right]{\text{ / ALS}}_{\text{T}}\text{ × 100 }$$

where ALS_T_ is the total amount of ALS added during preparation of the SLNs

ALS_S_ is the amount of ALS in the supernatant after the centrifugation

ALS_P_ is the amount of ALS in the purification medium

### Scanning electron microscopic (SEM) analysis

The morphologies of the prepared SLNs were characterized using a scanning electron microscope. According to the results of particle size and EE%, the optimum ALS-SLN formula was subjected for SEM examination. The samples of ALS-SLN were reconstituted with deionized water and spread over carbon tape. Samples were then dried in a vacuum, coated with gold and examined under the electron microscope to determine the surface morphology.

### Determination of in vitro gastro-resistance

To evaluate the enteric nature of eudragit coated ALS-SLN, the release of ALS from a certain amount of SLNs containing 10 mg of ALS was assessed in 0.1 N hydrochloric acid (pH 1.2) as a release media. The test was performed according to the method described previously by Hosny et al. 2013 [20].

### In vitro drug release Study

The in vitro release profile for ALS from eudragit-coated SLNs was evaluated in phosphate buffer, pH 7.4. ALS-SLNs, equivalent to 10 mg of ALS were suspended in 1 mL of phosphate buffer, pH 7.4, in a glass cylinder covered by 0.1 μm semipermeable membrane and suspended in 250 ml of phosphate buffer pH 7.4, and rotated at 75 rpm. A 5ml samples of dissolution medium was withdrawn after 5, 10, 15, 30, 45, 60, 90, 120, 150, and 180 minutes and assayed for ALS at 333 nm.

### Stability Study

To ascertain stability of formulations for future commercial viability, stability studies were carried out for optimum formulation. The formula was stored for a period of 30, 60 and 90 days at 4.0±1°C and 25±2°C in screw-capped, amber-coloured small glass bottles. Particle size and residual drug content were analysed and assessed by means of ANOVA tests, which showed the existence of significant or nonsignificant differences (p < 0.05) between the particle size and EE% before and after storage.

### In vivo pharmacokinetics study

Twelve healthy albino male rabbits (2–2.5 kg) were obtained from central animal facility of King Abdulaziz University, and divided into two groups, with six animals each. Animal were maintained under standard laboratory conditions of 12 hours light/dark cycle at 23±2°C, and given pellet diet with water ad libitum and fasted for 12 hours before and during the experiment. The in vivo animal studies protocol was revised and approved by the ethical committee at King Abdulaziz University, Jeddah, Saudi Arabia. An oral formula of Eudragit-coated freeze-dried ALS-SLN was given to one group, and 10-mg Fosamax^®^ tablets (Merck & Co., Inc. USA) were given to the other group at a dose of 1 mg/Kg for each animal group. Animals were continuously monitored during the first 12 hr, then checked at 24 h after injection. Animal were placed in individual restrainers and lidocaine 10% was sprayed on the ear to provide sufficient local anesthesia, then a polyethylene catheter (0.56 mm i.d., 0.98 mm o.d.) was inserted into the marginal ear vein of each rabbit for collection of blood samples. Blood samples were collected before the drugs were administered and at different time intervals of 0.5, 0.75, 1, 1.5, 2.5, 3.5, 5, 6, 12, and 24 hours. Serum samples were then analysed by HPLC. Our protocol indicated that animals should euthanized in cases of excessive signs of distress, limited water consumption, lethargy, or lameness. No mortality was detected during the whole of experiment. After performing the test, animals were euthanized by injection of beuthanasia (0.5ml/2kg) after anaesthesia with isofluorane. Pharmacokinetic parameters were calculated and are presented as the mean ± S.D. C_max_ and T_max_ after oral administration were calculated directly from the plasma concentration-time curve using the WinNonlin^™^ Nonlinear Estimation Program. One-way analysis of variance (ANOVA) was employed to assess the significance of the difference between the tested ALS-SLN formulation and the reference at a level of p≤ 0.05 using the SPSS program [21]. AUC_0-24_ was calculated using the linear trapezoidal rule [22]. AUC_0-∞_ was determined by adding the last measured plasma concentration divided by the elimination rate constant (K_el_) to AUC_0-24_. The relative bioavailability (BA_R_) of enteric coated NLS was calculated using the following formula ${\text{BA}}_{\text{R}}=\;\left[{\text{AUC}}_{\text{SLN}}{\text{x Dose}}_{\text{tablet}}\right]\text{ / }\left[{\text{AUC}}_{\text{tablet}}{\text{x Dose}}_{\text{SLN}}\right]\;$ [23]

## Results

### Screening of the lipid phase

For the selection of a lipid core, the solubility of ALS in lipids was evaluated by measuring the ratio of the amount of ALS in the lipid phase to the amount of ALS in the aqueous phase. The results obtained were 0.25±0.05, 0.28±0.11, 0.32±0.09, and 0.45±0.13 for compritol 888 ATO, Precirol ATO5, stearic acid, and glyceryl monostearate, respectively. The highest partition coefficient was observed with glyceryl monostearate, which was selected as a core lipid for the preparation of SLNs.

### Fitting of model and measurement of model suitability

A total of 15 experimental runs with three variables and three levels was conducted using different combinations of independent variables, including percentage of lipid (glyceryl monostearate), percentage of surfactant (Lutrol 68), and sonication time, using the Box-Behnken design in order to study their effect on the dependent variables, which were the particle size and EE%.

For the fitting of the model, two different tests, namely, sequential model sum of squares and model summary statistics, were performed by fitting various polynomial models, namely, linear, interactive (2FI), and quadratic models, to the experimental data.

It was observed from the sequential model sum of squares test presented in Tables 2 and 3 that the quadratic model was significant (p-value ≤ 0.01) at a corresponding F value of 12.41 and 17.79 for particle size and EE%, respectively.

**Table 2:** Fit summary model for particle size.

**Sequential Model Sum of Squares for Particle size**						
**Source**	**Sum of Squares**	**df**	**Mean Square**	**F Value**	**p-value (Prob> F)**	**Action**
**Mean vs Total**	3.757E+005	1	3.757E+005			
**Linear vs Mean**	13239.75	3	4413.25	4.22	0.0326	
**2FI vs Linear**	4558.50	3	1519.50	1.75	0.2345	
**Quadratic vs 2FI**	6131.43	3	2043.81	12.41	0.0094	Suggested
**Cubic vs Quadratic**	287.25	3	95.75	0.36	0.7939	Aliased
**Residual**	536.00	2	268.00			
**Total**	4.005E+005	15	26698.53			
**Model Summary Statistics for particle size**						
**Source**	**Std. Dev.**	**R-Squared**	**Adjusted R-Squared**	**Predicted R-Squared**	**PRESS**	**Action**
**Linear**	32.35	0.5349	0.4080	0.0276	24069.58	
**2FI**	29.48	0.7190	0.5083	-0.3966	34569.50	
**Quadratic**	12.83	0.9667	0.9069	0.7656	5802.00	Suggested

**Table 3:** Fit summary model for EE%.

**Sequential Model Sum of Squares for EE%**						
**Source**	**Sum of Squares**	**df**	**Mean Square**	**F Value**	**p-value (Prob> F)**	**Action**
**Mean vs Total**	81232.32	1	81232.32			
**Linear vs Mean**	3.55	3	1.52	10.36	0.0153	
**2FI vs Linear**	1.50	3	0.50	0.42	0.7440	
**Quadratic vs 2FI**	8.70	3	2.90	17.79	0.0042	Suggested
**Cubic vs Quadratic**	0.82	3	0.27			Aliased
**Residual**	0.000	2	0.000			
**Total**	81547.89	15	5436.53			
**Model Summary Statistics for EE%**						
**Source**	**Std. Dev.**	**R-Squared**	**Adjusted R-Squared**	**Predicted R-Squared**	**PRESS**	**Action**
**Linear**	1.00	0.9651	0.9556	0.9414	18.48	
**2FI**	1.09	0.9698	0.9472	0.9075	29.19	
**Quadratic**	0.40	0.9974	0.9928	0.9586	13.05	Suggested

### Polynomial equation and Statistical analysis

Data obtained from experimental runs were fitted to quadratic models for responses. Multiple regression analyses were performed to fit models to the experimental data in order to generate second-order polynomial equations. The final quadratic equation generated by the Design-Expert software was given as follows:

$$\text{Particle size }\left(\text{nm}\right)\;=\;+\text{154}.00\;+\text{ 25}.\text{36A }+\text{ 9}.\text{12B }–\text{ 3}0.\text{25C }-\;0.\text{14AB }+\text{ 33}.\text{75AC }-\;0.\text{75BC }+\text{25}.{\text{25A}}^{\text{2}}+\text{1}0.{\text{75B}}^{\text{2}}-\text{28}.00{\text{ C}}^{\text{2}}$$

$$\text{EE }\left(\%\right)\;=\;+\text{74}.\text{9}0\;+\text{ 5}.\text{67A }-\text{ 2}.\text{41B }–\;0.\text{29C }-\;0.\text{16AB }-\;0.\text{17AC }+\;0.\text{59BC }-0.{\text{41A}}^{\text{2}}-\text{1}.{\text{42B}}^{\text{2}}-0.{\text{62C}}^{\text{2}}$$

The statistical analysis was performed using ANOVA, and the results are presented in Tables 4 and 5. The significance of the quadratic model and regression coefficient were evaluated by their corresponding F and p-values. The p-value is used to check the significance of each regression coefficient and the interaction strength between variables. The higher the significance is, the better is the degree of correlation between the observed and predicted values.

**Table 4:** Analysis of variance of particle size (nm).

Analysis of variance of particle size						
Source	Sum of Squares	df	Mean Square	F value	p-value (Prob>F)	Significance
**Model**	23929.68	9	2658.85	16.15	< 0.005	significant
***A-Glyceryl monostearate conc*.**	*5253*.*12*	*1*	*5253*.*12*	*31*.*90*	*0*.*0024* ^b^	significant
***B-Lutrol 68 conc*.**	*666*.*12*	*1*	*666*.*12*	*4*.*05*	*0*.*1005*	
***C-Sonication time***	*7320*.*50*	*1*	*7320*.*50*	*44*.*46*	*0*.*0011* ^b^	significant
***AB***	*0*.*000*	*1*	*0*.*000*	*0*.*000*	*1*.*0000*	
***AC***	*4556*.*25*	*1*	*4556*.*25*	*27*.*67*	*0*.*0033* ^b^	significant
***BC***	*2*.*25*	*1*	*2*.*25*	*0*.*014*	*0*.*9115*	
***A***^***2***^	*2354*.*08*	*1*	*2354*.*08*	*14*.*30*	*0*.*0129*	
***B***^***2***^	*426*.*69*	*1*	*426*.*69*	*2*.*59*	*0*.*1684*	
***C***^***2***^	*3894*.*77*	*1*	*3894*.*77*	*22*.*58*	*0*.*0045*	significant

^a^: p < 0.0001

^b^: 0.0001 ≤ p < 0.05

**Table 5:** Analysis of variance of EE%.

Analysis of variance of EE%						
Source	Sum of Squares	df	Mean Square	F Value	p-value (Prob> F)	Significance
**Model**	314.75	9	34.97	214.39	< 0.0001	significant
***A-Glyceryl monostearate conc*.**	*257*.*53*	*1*	*257*.*53*	*1578*.*74*	*< 0*.*0001* ^a^	significant
***B-Lutrol 68 conc*.**	*46*.*32*	*1*	*46*.*32*	*283*.*96*	*< 0*.*0001* ^a^	significant
***C-Sonication time***	*0*.*70*	*1*	*0*.*70*	*4*.*27*	*0*.*0937*	
***AB***	*0*.*11*	*1*	*0*.*11*	*0*.*65*	*0*.*4575*	
***AC***	*0*.*000*	*1*	*0*.*000*	*0*.*000*	*1*.*0000*	
***BC***	*1*.*39*	*1*	*1*.*39*	*8*.*54*	*0*.*0330*	
***A***^***2***^	*0*.*62*	*1*	*0*.*62*	*3*.*83*	*0*.*1078*	
***B***^***2***^	*7*.*46*	*1*	*7*.*46*	*45*.*72*	*0*.*0011* ^b^	significant
***C***^***2***^	*1*.*44*	*1*	*1*.*44*	*8*.*81*	*0*.*0312*	

^a^: p < 0.0001

^b^: 0.0001 ≤ p < 0.05

**Fig 1:**
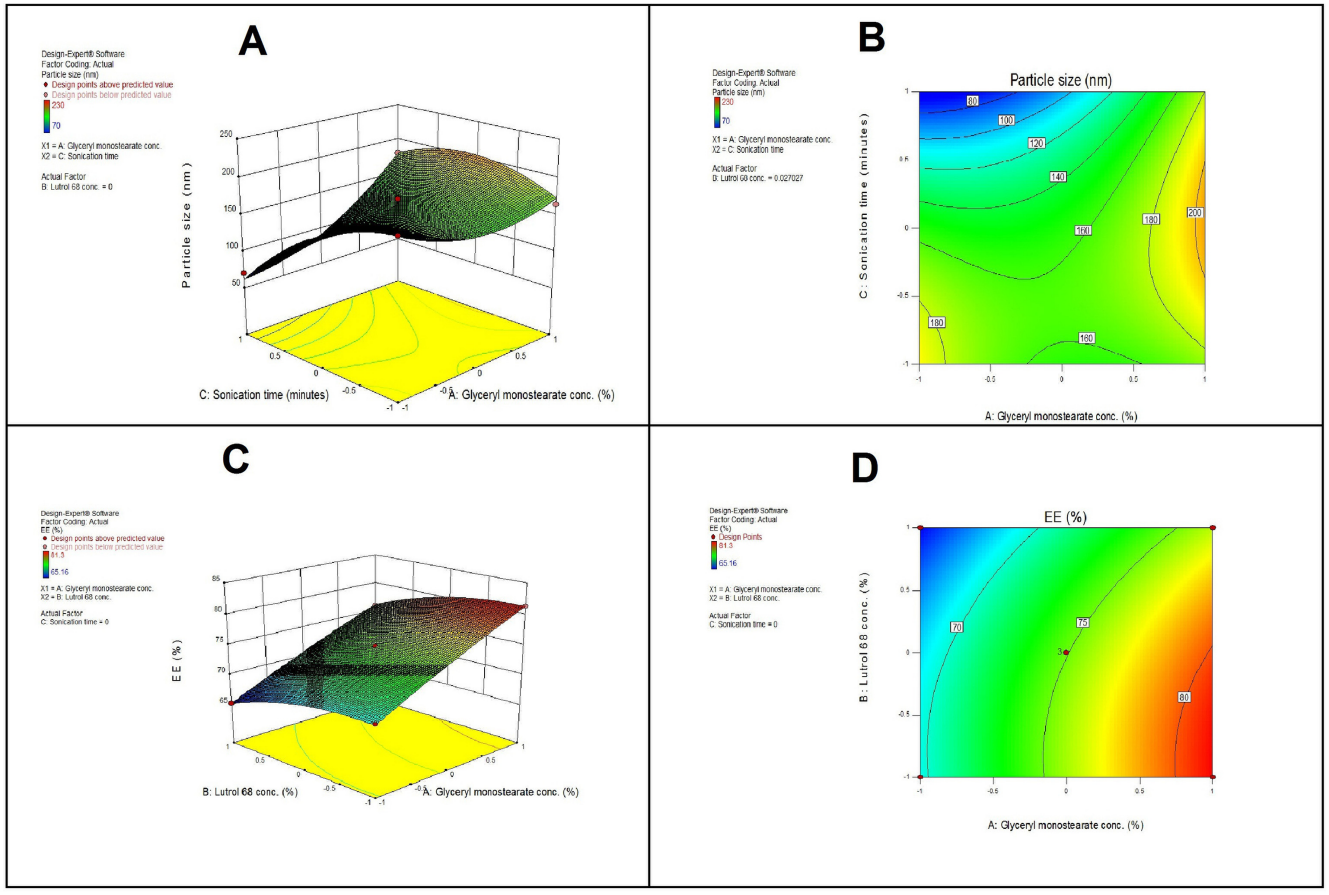
A-Response surface plot (3D) showing the effect of X_1_ and X_3_ on Y_1._, B-Contour plot showing the relationship between various levels of two variables to attain fixed values of Y_1_., C-Response surface plot (3D) showing the effect of X_1_ and X_2_ on Y_2._, and D-Contour plot showing the relationship between various levels of two variables to attain fixed values of Y_2_.

The relationship between the factors and responses can also understood by plotting the response surface and contour for the estimated effects Fig 1.

### Optimization using the desirability functions

**Fig 2:**
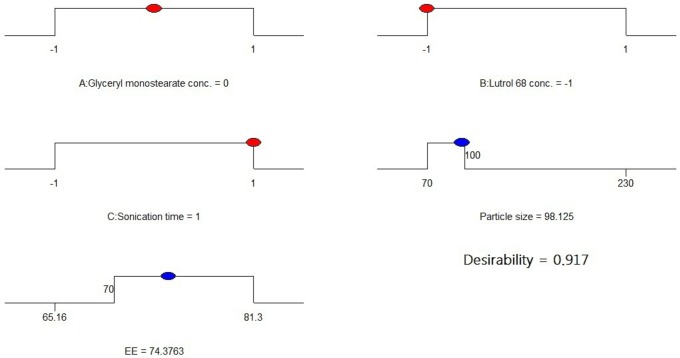
Desirability ramp for optimization.

Derringer’s desired function methodology was used to optimize the particle size and EE% of the prepared SLNs. The results Fig 2 showed that the optimum conditions were glyceryl monostearate at 30% w/w, Lutrol 68 at 5% w/w, and a sonication time of 8 minutes. Under these conditions, the predicted particle size is 98 nm and the entrapment efficiency is 74% with a desirability value of 0.917. Based on the above observation, experimental run 11 was considered to be the optimized formulation.

### Morphology and size analysis of the optimized ALS-SLN formula

**Fig 3:**
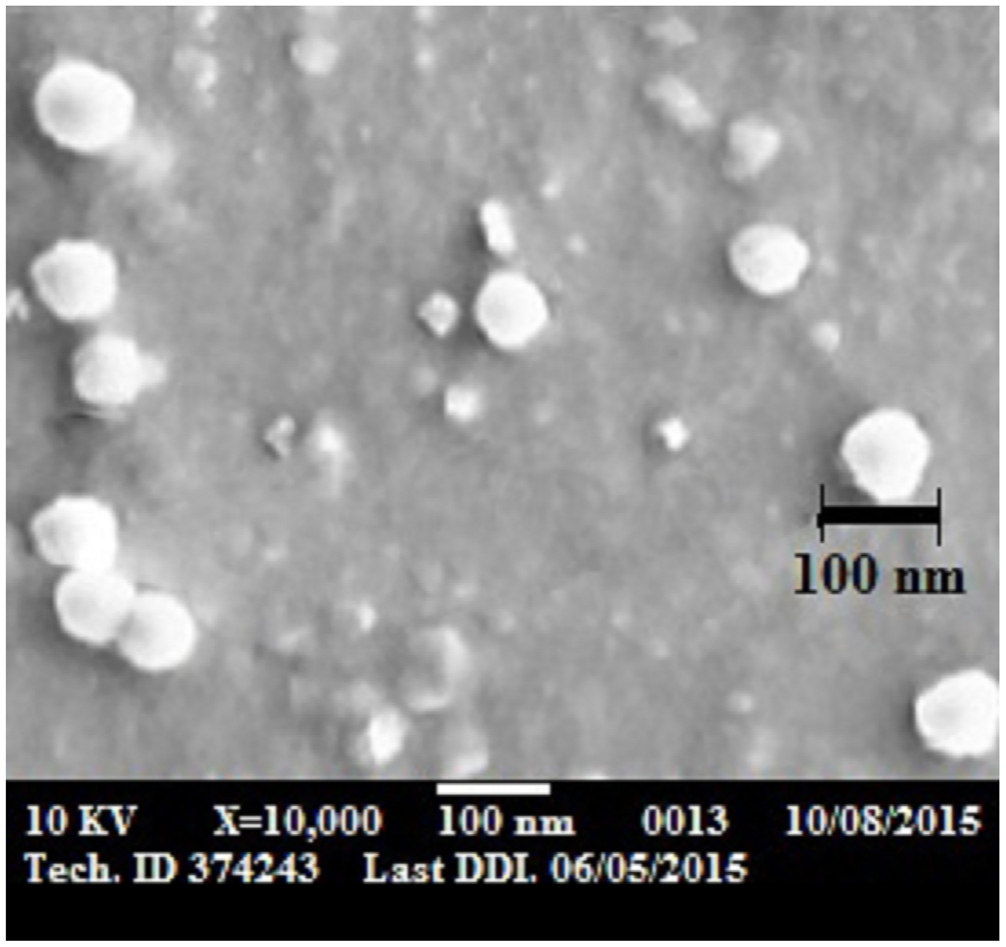
SEM image of freeze dried optimized formula of ALS-SLN.

Fig 3 shows the SEM for freeze dried ALS-SLN optimized formula. The SEM revealed the presence of homogenous, well-identified spheres, and they exist in a dispersed pattern, with a size of approximately 100 nm. This confirms the results of the particle size analysis using a Malvern Zetasizer instrument (#Nano ZS 4800, UK) at 25°C which indicated that the size for optimized formula was 99 ±4 nm.

### Stability study

After storage of the optimized formula of ALS-SLNs for a specified time period of 30, 60, and 90 days at 4.0±1°C and at 25±2°C, particle size and EE% of the optimized formula were determined before and after storage by the same methods used during preparation. An initial drug content of 100% was taken for each formulation, and the % residual drug content was calculated. By keeping the initial drug content at 100%, the determination of the % residual drug in the SLNs showed that 1–2% of the drug was lost from the formulation within 90 days when stored at 4.0±1°C and approximately 3% of the drug was lost from those stored at 25±2°C. This could be due to more leaching of the drug from SLNs at room temperature than at 4°C. Regarding the particle size, there was no significant change after storage at 4.0±1°C, but at 25±2°C, there was a slight increase in particle size of the SLNs (from 96 nm to 106 nm), which could be due to aggregation of SLN at this temperature. Thus, according to this finding, we can conclude that for better stability, the formulation should be stored under refrigerated conditions, i.e., 4.0±1°C.

### In vitro drug release

**Fig 4:**
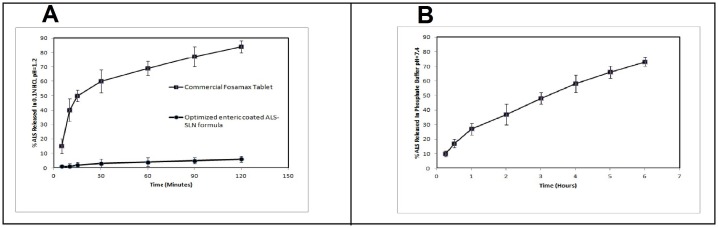
**A-** In vitro release of ALS from optimized eudragit-coated SLN and from a commercial Fosamax tablet in 0.1 N HCl, pH 1.2. **B-** In vitro release of ALS from optimized eudragit-coated SLN in phosphate-buffered saline, pH 7.4.

Fig 4 shows the in vitro release profile of ALS from the optimized ALS-SLN formula at pH 1.2 in order to evaluate the enteric nature of the coated SLNs. The percentages of ALS released within 2 h were only 5% and 85% from ALS-SLN and from Fosamax^®^ tablets, respectively. This ensures the gastro resistance of the enteric coated formula. Fig 4 shows the in vitro release profile of ALS from the optimized ALS-SLN formula in phosphate-buffered saline at pH 7.4.

### In vivo Pharmacokinetics study

The plasma drug concentration of the optimized ALS-SLN formulation and from the Fosamax^®^ tablet in rabbits after oral administration and the pharmacokinetics parameters are tabulated in Table 6. At each sampling time, the ALS-SLN plasma concentration was significantly higher for rabbits treated with ATC-SLN than for those treated with reference tablets. The C_max_ values of ALS in SLN was higher (16.54±3.12 ng mL^-1^) than that obtained with the commercial tablet (4.92±1.02 ng mL−1) after oral administration. It was also observed that the absorption of ALS was rapid and reached its peak plasma concentration in 0.75 h ±0.09 with the commercial tablet, whereas the mean T_max_ for the tested SLN was 4.5±0.5 h. The mean AUC_0-∞_ for the Fosamax^®^ tablet was significantly different compared with the SLN.

**Table 6:** Pharmacokinetic parameters after oral administration of optimized Eudragit-coated ALS-SLN formula and Fosamax^®^ Tablet (n = 6). The mean difference is significant at the 0.05 level.

Formulation	T_max_ (hr)	C_max_ (ng/ml)	AUC_0-t_ (ng.hr/ml)	K (hr^-1^)	AUC_0-∞_ (ng.hr/ml)
**Optimized Eudragit coated ALS-SLN formula**	4.5±0.52	16.54±3.12	2173±216.4	0.203±0.06	3781.5±296.4
**Fosamax**^**®**^	0.75 ±0.13	4.92±1.02	291.6±28.14	0.638±0.17	511.4±38.21

## Discussion

The Box-Behnken experimental design is one of the response surface models used to hit the target, reduce variability in the experiment, maximize/minimize a response that increases the production yield or decreases the amount of waste. For the fitting of the model, two different tests, namely, sequential model sum of squares and model summary statistics, were performed. Adj. R2 and PRESS values were compared, and the main focus of the experimental design was to maximize the Adj. R^2^ and Pred. R^2^ values. Unlike the R^2^ value, a low PRESS value indicated adequate fitting of the model. The quadratic model indicated that the highest values of R^2^ for particle size and EE%, respectively. At the same time, it demonstrated low PRESS values compared to the other model. The higher R^2^ value is an indication of a good correlation between actual values and predicted values, which in turn better represents the suitability of the response surface model [24]. Furthermore, the difference between Adj. R^2^ and Pred. R^2^ of less than 0.2 (in the present study, the difference was 0.1413 and 0.0342 for particle size and EE%, respectively) demonstrates a high degree of correlation between the actual values and predicted values obtained from the regression model [25]. From the above findings, it was concluded that the quadratic model was the best fit model for the responses.

Regarding the results of particle size, the quadratic model was significant with an F value of 16.15 (p< 0.005). For the given p values, two linear coefficients (A and C), one interactive coefficient (AC) and one quadratic coefficient (C^2^) suggested the pattern of interactions between the tested independent variables. The degree of influence of individual factors on particle size indicated that ultrasonication time had a huge effect on particle size and that particle size was dramatically decreased as the sonication time increased. This could be because sonication is responsible for making the final particle size in SLNs, which broke the coarse emulsion drops into nanoemulsion droplets [26]. Yosra et al. (2011) have also reported that the particle size of topical sildenafil citrate SLNs was dependent on the sonication time [27].

The positive effect of the glyceryl monostearate concentration on particle size could be due to the increased viscosity of the system during preparation of the high lipid concentration, which decreases the efficiency of sonication and particle size reduction. Additionally, this could be due to the decrease in the emulsifying efficiency and the increase in particle agglomeration at high lipid concentrations.

Regarding the effect of lutrol 68 surfactant on particle size, a non-significant positive effect was observed. This result agrees with the work of Das et al. (2012), who also reported similar results of an increase in particle size for surfactants with higher hydrophilic lipophilic balance (HLB) values, such as the use of Poloxamer 188, in comparison to a low-HLB-value surfactant [28].

Regarding the results of entrapment efficiency, the quadratic model was significant with an F value of 214.39 (p< 0.0001). The degree of influence of individual factors on EE% indicated that by increasing the concentration of glyceryl monostearate, the EE% increased. This may be because a higher lipid concentration provided more space to accommodate an excessive amount of drug during SLN preparation. Additionally, this may due to the high lipid concentration reducing the amount of drug escaping into the external phase, which accounts for an increase in EE. These results agreed with the work of Shah et al. (2007) [29].

Regarding the effect of Lutrol 68 surfactant on EE%, it was noted that a higher EE% was attained at a low surfactant concentration. Then, by increasing the surfactant concentration, the EE% decreased. This could be explained by the partitioning phenomenon, which states that at a high surfactant concentration level in the external phase, the solubility of the drug in the external phase increases. This increases the partitioning of the drug from the internal to the external phase of the medium leading to a decrease in entrapment efficiency, another reason that leaded to inability of EE% to be more than 74% is the higher hydrophilicity of the drug which aided in partitioning and escaping of drug from the SLNs to the external aqueous phase [30].

From the results shown in Fig 1A, at a fixed percent of lutrol 68, while decreasing the percentage of glyceryl monostearate and increasing the sonication time, the particle size decreased to 70 nm. However, using a high level of X_1_ while decreasing X_3_ results in increases in the Y_1_ to more than 150 nm. The contour plot (Fig 1B) suggests the exact percentage of X_1_ and X_3_ at which the particle size is minimized at a fixed level of X_2_. From the figure, we can conclude that using X_1_ at low levels, along with high levels of X_3,_ can produces a SLN that has a particle size within 70 to 100 nm.

The effect of X_1_ and X_2_ on the EE% (Y_2_) is displayed in a response surface plot (Fig 1C). At a fixed level of X_3_, increasing X_1_ while decreasing X_2_ increases Y_2_ to 81%; using a low level of X_1_ while increasing X_2_ decreases Y_2_ to 65%. Additionally, the contour plot (Fig 1D) suggests the exact percent of X_1_ and X_2_ at which the SLN encapsulated the maximum amount of the drug. As seen in the figure, using X_1_ at a high level with a low X_2_ can encapsulate the maximum amount of drug.

The results of in vitro release ensured the gastro resistant nature of the optimized enteric coated formula as 5% only released within 2hr in acidic medium. While approximately 10% ALS was released within 15 minutes in basic medium, which indicates the rapid dissolution of the enteric coating material in pH 7.4. The release at this percent could be attributed to the presence of free drug (un-entrapped) on the surface of the SLNs and just beneath the eudragit coat. After that, nearly 73% of ALS was released within 6 h in a homogeneous and sustained form, which indicated that the ALS homogeneously dispersed in the lipid matrix, the release was found to follow Weibull and Higuchi equations. From the previous results it can be concluded that the optimized formula succeeded in reaching the aim of this work, which was the prevention of the release in acidic medium in order to avoid the adverse effects.

The results of pharmacokinetic study confirmed that the bioavailability of ALS was enhanced more than 7.4-fold when formulated as enteric coated SLNs. The delay in T_max_ in the case of SLN is attributed to the efficient coating with eudragit S100 that hinders the release of ALS in the stomach (acidic pH). The enhancement in bioavailability, more than 7.4-fold, could be attributed to the size of the SLNs (approximately 100 nm), which has the advantages of nanoparticles, which improve the adhesion to and absorption into intestinal epithelial cells [31]. In addition, solid lipid nanoparticles may promote the uptake by M-cells in the Peyer’s patches and increase the absorption through the lymphatic pathway. Furthermore, permeation of intact SLNs through the intestinal epithelial pathway is considered to be a different potential mechanism [32].

## Conclusion

Formulation of ALS as eudragit-coated solid lipid nanoparticles as a novel drug delivery system provided the maximum gastro-resistant release to eliminate the undesired ALS adverse effects. The oral bioavailability of ALS was enhanced by more than 7.4-fold in relation to the commercially available product. The results indicated that the effect of independent variables on particle size and entrapment efficiency can be predicted and precisely interpreted using the Box-Behnken design employed Design expert^®^ software. Using Derringer's desirability as a functional tool for optimization, the highest EE% value of 74.3% and the smallest size value of 98 nm were predicted under optimum preparation conditions, with a desirability value of 0.917. The improved ALS formula could reduce the major drawbacks of conventionally used tablets and allow osteoporotic patients to tolerate the drug easily at any time in any position without fear of oesophageal inflammation and/or bleeding.
